# Cefadroxil-Mupirocin Integrated Electrospun Nanofiber Films for Burn Wound Therapy

**DOI:** 10.2174/0115672018374558250607134659

**Published:** 2025-06-18

**Authors:** Saman Rashid, Munaza Ijaz, Sana Rafique, Haya Yasin, Mahnoor Mushtaq, Abida Kalsoom Khan, Madiha Khan, Bushra Nasir, Ghulam Murtaza

**Affiliations:** 1 Department of Pharmaceutics, Faculty of Pharmacy, Bahauddin Zakariya University, Multan, Pakistan;; 2 Department of Microbiology, University of Central Punjab, Lahore, Pakistan;; 3 Department of Chemistry, COMSATS University of Islamabad, Abbottabad Campus, Abbottabad 22060, Pakistan;; 4 Department of Pharmaceutical Sciences, College of Pharmacy and Health Sciences, Ajman University, Ajman 346, United Arab Emirates;; 5 Department of Pharmacy, COMSATS University of Islamabad, Lahore Campus, Lahore 54000, Pakistan

**Keywords:** Polyvinyl alcohol, chitosan, drug release, antibacterial activity, nanofiber, burn wound

## Abstract

**Introduction:**

This study aims to fabricate dual drug-loaded nanofibrous films made from polyvinyl alcohol (PVA) and chitosan, incorporating cefadroxil and mupirocin to meet the critical needs of burn wound care.

**Methods:**

Electrospinning was utilized to fabricate cefadroxil- and mupirocin-loaded polyvinyl alcohol PVA/Chitosan nanofibers. Characterization of structural and morphological properties of these nanofibers was done through Fourier Transform IR Spectroscopy, Scanning Electron Microscopy, Thermal analysis by TGA, and XRD spectroscopy. The kinetic profiles of the drug release mechanisms were considered to determine the release of cefadroxil and mupirocin. Antibacterial activity was determined against the bacteria *Staphylococcus aureus* and *Pseudomonas aeruginosa*, while the wound healing efficacy was tested in a rabbit model using full-thickness wounds.

**Results:**

SEM analysis demonstrated the formation of uniform and smooth nanofibers possessing a well-defined morphology. FTIR spectroscopy confirmed the successful incorporation of cefadroxil and mupirocin into the PVA/Chitosan matrix. TGA analysis indicated the thermal stability of the nanofibers, while XRD results suggested that the drugs were either molecularly dispersed or in an amorphous state within the biopolymeric blend. Drug release studies showed distinct profiles, with an initial burst release followed by sustained drug release. Over 80% of mupirocin was released within the first 2 hours, while cefadroxil exhibited a cumulative release exceeding 60%. Antibacterial assays showed significant inhibition zones, with the largest being **20 mm against *Staphylococcus aureus***. *In vivo* studies utilizing a full-thickness rabbit wound model revealed that the drug-loaded nanofibers accelerated wound contraction, achieving approximately 90% closure by day 17, compared to less than 70% for the control.

**Discussion:**

The dual drug-loaded PVA/Chitosan nanofiber films demonstrated excellent antibacterial efficacy and improved wound healing, indicating their therapeutic potential for burn wound management. The combination of cefadroxil and mupirocin within the nanofiber matrix enabled rapid initial drug release followed by sustained delivery, contributing to effective infection control and tissue regeneration.

**Conclusion:**

The study demonstrates that cefadroxil-mupirocin nanofiber films provide superior antibacterial activity and faster wound healing rates, highlighting their potential in advanced burn wound management.

## 1 INTRODUCTION

Nanofibers are novel materials that have characteristics like high surface area to volume ratio, nanometer size, porous nature, high drug encapsulation efficiency, and selective releasing nature [1], which makes them potential candidates for several biomedical applications [2]. These fibers, generally created by techniques such as electrospinning, are characterized by mechanical flexibility, controllable porosity, and functional surface [3]. Nanofibers have been identified as potential drug carriers and tissue engineering scaffolds because of their resemblance to the ECM, cell affinity, and controlled drug-releasing properties [4].

In the field of drug delivery, nanofibers provide several advantages. Their high surface area facilitates efficient drug loading, while their interconnected porous networks enable controlled and sustained delivery of the drugs. Additionally, nanofibers can be engineered to encapsulate multiple drugs, offering the possibility of combinatorial therapy to enhance treatment efficacy [5]. The ability to incorporate both hydrophilic and hydrophobic drugs further broadens their application scope. By delivering drugs directly to the target site, nanofiber-based systems improve bioavailability, reduce systemic side effects, and ensure localized treatment, making them an ideal choice for wound healing applications [6].

Burn wounds, in particular, pose significant challenges due to their susceptibility to bacterial infections, prolonged healing times, and the need for specialized care. An ideal wound dressing should fulfil multiple criteria, such as maintaining a moist environment, protecting the wound from microbial contamination, absorbing exudates, and promoting tissue regeneration [7]. Electrospun nanofibrous scaffolds have shown remarkable potential in addressing these needs. Their ECM-like structure supports cellular activities, while their ability to be loaded with antimicrobial agents helps in preventing infections commonly associated with burn injuries. The sustained release of multiple antibiotics at the target site in a controlled manner at the expense of minimal toxicity to human cells has been made possible by the biomaterial-based drug delivery system in the form of nanofibers [8].

Recent technologies in polymer science have allowed the synthesis of biopolymer nanofibers with greater biocompatibility and functionality [9]. Polyvinyl alcohol (PVA), which is a synthetic polymer, and chitosan, a natural biopolymer derived from chitin, are extensively applied in biomedical applications. PVA is hydrophilic, mechanically robust, and forms stable nanofibrous structures [10], while chitosan is inherently antibacterial, biodegradable, and promotes wound healing [11]. Pure chitosan is weak and more susceptible to swelling unless stabilized, *e.g.,* crosslinking with another polymer or crosslinking agent such as montmorillonite. However, to reduce the crystallinity of chitosan and to produce uniform ultrafine nanofibers PVA blend is used at the weight ratio of 50/50. Many researchers studied the chitosan with AgNP composite nanofibers fabrication that has synergistic effects and improved functionality for antibacterial efficiency [11-13]. As a result of the high level of resistance of microbes at the wound site to antibiotics, the use of one antibiotic is becoming less effective; therefore, more attention has been focused on the use of multiple antibiotics to fight drug-resistant bacteria works through synergism [14]. Furthermore, combining two antibiotics can help limit bacterial resistance by producing a synergistic effect, leading to improved wound healing through increased production of growth factors and cytokines [15].

Based on these principles, this study focuses on the fabrication of dual drug-loaded electrospun nanofibers incorporating cefadroxil and mupirocin within a PVA/Chitosan matrix. Cefadroxil, a first-generation cephalosporin, provides broad-spectrum antibacterial activity [16], while mupirocin is active against gram-positive bacteria such as *Staphylococcus aureus* [17], a Gram-negative bacteria [18]. Previous works documented its efficiency in the wound healing process and its ability to stimulate the secretion of TNF-α in macrophages at the early stages of wound healing, leading to the inflammatory phase. Mupirocin was attributed to its ability to enhance the healing of wounds [19]. Together, these drugs address a wide range of bacterial infections commonly associated with burn wounds.

This study aims to address the limitations of existing wound care solutions by leveraging the unique properties of nanofibers and the combined efficacy of cefadroxil and mupirocin (CFX-MUP). The studies highlight that PVA/Chitosan-based nanofibers could be the potential next-generation wound dressing for efficient management of burn wounds.

## 2 MATERIALS AND METHODS

### 2.1 Materials

Sigma Aldrich (International Laboratory, USA) provided montmorillonite (MMT), polyvinyl alcohol (PVA, MW = 72,000) and Chitosan. Raw powder of both drugs, cefadroxil, and mupirocin, were purchased from VWR Chemicals, Lahore, Pakistan. Polymeric solution preparation involves the use of deionized water as a solvent.

### 2.2 Solution Preparation for Electrospinning Process

The polymer solution was prepared by dissolving 1.7g of PVA in 12 mL deionized water under vigorous stirring to obtain 10% w/v solution. Chitosan solution (CS) (2% by weight) was prepared by dissolving chitosan in deionized water containing 1% acetic acid. Each polymer solution was sonicated, stirred and offered heat treatment with a temperature range of 50-60°C for 12 hours. The polymer ratios were kept constant in all formulations upon varying the concentration of drugs. Both the drugs were dissolved separately in deionized water and added into the total polymeric blend along with the 1% MMT solution.

### 2.3 Fabrication of Nanofiber Films by Electrospinning

**Fig. (1):**
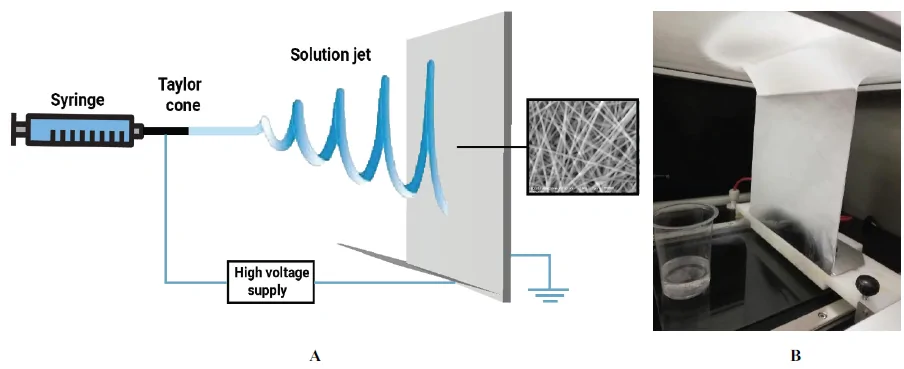
(**A**). Principle of the electrospinning process. (**B**). Nanofiber sheet.

For the setup of an electrospinning machine (Fig. **1A** and **B**), the solution was loaded in a 10 mL electrospinning syringe. The parameters for the electrospinning process were maintained at the ideal conditions. The syringe was obtained from BD, Pakistan with a diameter of 14.43 mm. The polymer solution was then electrospun using a FLUIDNATEK LE-10 electrospinning instrument at a flow rate of 0.30 – 0.60 mL/h. As the instrument voltage was gradually increased, the solution was charged, and the Taylor cone was observed at around 15 kV, which suggests that the electrospinning process was initiated [20]. The polymeric solution was deposited on the shiny metallic collector plate that had been coated with aluminum foil and formed thin fibers. All six formulations were electrospun using the same process parameters mentioned above (Table **1**).

### 2.4 Chemical Characterization of CFX-MUP loaded PVA/CS Nanofiber Films

#### 2.4.1 Surface Morphology by Scanning Electron Microscopy

The surface characteristics and topology of electrospun nanofibers loaded with CFX-MUP were analyzed using Joel, JSM 6400F SEM. Further, the morphology of the sample can be clearly analyzed by the 3D structure through SEM analysis [21]. The principle of the SEM analysis involves the use of high electron beams on the surface of the material. When the highly concentrated electron beams focus on the sample surface, they produce signals [22], which give useful information on the surface topography and also the morphology of samples. The diameter size distribution of the nanofibrous mat was determined by Image J software by measuring individual fibers randomly.

#### 2.4.2 FTIR Analysis for Functional-groups Identification

FTIR analysis allows for obtaining important information regarding the nature of bonding. FT/IR-6600 type A was employed in collecting the spectra of CFX-MUP incorporated electrospun polymeric nanofibers in the specified region of 4000-499 cm-1 while addressing the intermolecular aspect [22]. The functional group analysis is done qualitatively as well as quantitatively with the help of FT-IR spectroscopy, which predictably identifies the specific vibrations that are associated with the chemical bonds of a sample. FTIR spectroscopy causes vibrational transitions in its sample as it transmits IR radiations through the sample [23]. Absorption of IR radiation occurs when the radiation frequency matches the bond absorption frequency and the spectrum is taken.

#### 2.4.3 Thermal Gravimetric Analysis (TGA)

The thermal stability of both CFX-MUP-loaded and blank electrospun nanofibers was evaluated using a Q600 SDT thermogravimetric analyzer. The nanofiber samples were introduced into the nitrogen flow with an analytical and stable flow rate of 20 mL/min [24]. Thermal degradation studies of blank and CFX-MUP loaded nanofibers were performed by increasing the temperature at the rate of 10°C/min from 20°C to 500°C. The weight loss and temperature changes that occurred over time for CFX-MUP loaded and blank nanofibers were investigated. Thermal analysis of these nanofibers reveals various weight-loss regions at different temperatures [25].

#### 2.4.4 XRD Spectroscopy

The structural characterization of the nanofibers was done using XRD analysis. It differentiates between the structural morphology (crystalline or amorphous) of the nanofibrous Films [26]. Some key structural features of pure drugs, CFX-MUP-loaded nanofiber Films, and drug-free (MMM-1) NFs, such as the measurements of the degree of crystallinity, were determined by using a Diffractometer.

### 2.5 Pharmaceutical Research Studies

#### 2.5.1 Drug Solubility Study

To check the solubility of drug-loaded nanofibers, different organic and inorganic solvents were used, including distilled water, chloroform, DMF, DMSO, acetone, *etc*. To get rid of the excess amount of the drug, about 5 mg of both drugs were added into 5 mL of different solvents and were stirred at 37 rpm for 60 min. By using a spectrophotometer, absorbance was measured at 220 nm [27].

#### 2.5.2 Standard Curve

A standard stock solution (1000µg/mL) of pure mupirocin and cefadroxil was prepared by dissolving 100mg of the drug in 100 mL of deionized water. It was prepared in 50 v/v SBP (acetonitrile sodium phosphate buffer) at a pH of 6.4 [28]. The second dilution was made to 100µg/ml by preparing 1 mL stock solution and adding it to 10 mL deionized water. Various dilutions were prepared from the stock solution so as to obtain a working concentration of 40, 45, 50, 55, 60, 65, and 70 *µ*g/ml, which are to be used in absorbance measurements at the wavelength of λ=220 nm. Shimadzu, UV-1280, and Spectrophotometer were used to measure the absorbance. The calibration curve was plotted against various solution concentrations.

#### 2.5.3 In-vitro Drug Release Kinetics

To demonstrate the dissolution behavior of the fabricated bi-polymeric nanofibrous scaffold, 10 mg of the nanofibrous scaffold was dissolved in 30 mL of distilled water. Aliquots (2 mL) of dissolution solution were collected in a cuvette at different time intervals until 6 h. The release kinetics for incorporated drugs from the nanofiber films were measured and calculated by using a UV-visible spectrophotometer. The drug released (%) was determined by using the below-given equation:

Where mᵢ and m_t_ represent the amount of drug released at a time “t” and the total amount of drug loaded in the nanofiber sample.

In addition, drug release data was kinetically analyzed using equations 1-4, as stated below:


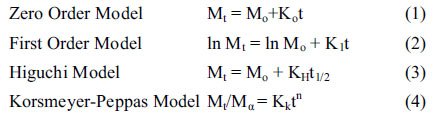


Where M_t_ represents the total amount of drug released at a specified time point, and M_o_ indicates the initial amount of drug in the formulation. K_o,_ K_1,_ K_H,_ and K_k_ are rate constants for zero order, first order, Higuchi, and Korsmeyer-Peppas models, respectively. In Equation 4, the n-value represents the slope of the Korsmeyer-Peppas plot [29].

#### 2.5.4 In-vivo Wound Healing Studies Using CFX-MUP-loaded Nanofibrous Films

The following study protocols were reviewed and approved by the COMSATS University Islamabad (CUI) Research Ethics Committee (Approval No. No. 897/CUI/PHM-2023). This study adheres to internationally accepted standards for animal research, following the 3Rs principle. The ARRIVE guidelines were employed for reporting experiments involving live animals, promoting ethical research practices. The *in-vivo* wound healing experiment was conducted by the Department of Pharmacy, CUI, Lahore Campus, Pakistan. This experiment was performed to evaluate and compare the efficacy of CFX-MUP-loaded electrospun nanofiber Films with the Quench ® commercial antibiotic ointment. For this purpose, three healthy rabbits (*n* = 3) were taken and divided into groups, *i.e.,* MMM-4 (CFX-MUP-loaded electrospun nanofibers), Quench cream, and a control group. Rabbits were anesthetized with xylene and ketamine hydrochloride. The rabbit skin was shaved by using the hair removing cream and disinfected with ethanol. Under an aseptic environment, two scars of the area of about 3-4 cm^2^ were created using a sterilized biopsy punch, and full thickness wound of about 1.5 cm was created. The first rabbit was treated with CFX-MUP-loaded electrospun nanofiber films, and meanwhile, the second rabbit was given the marketed standard ointment, Quench^®^. Whereas the third rabbit was considered as a control group and wounds were left with no medication. The wound healing studies were carried out for 21 days.

#### 2.5.5 Histopathological Studies

Histopathological examinations were conducted to determine the effectiveness of CFX-MUP-loaded electrospun nanofibers on local tissues. The tissues of rabbit skin were excised and fixed in 10% formalin. Paraffin-embedded skin tissues were sliced on glass slides, which were then stained with Hematoxylin and Eosin. Photomicrographs were captured under a high-resolution BX43, 40× microscope.

#### 2.5.6 Antimicrobial Activity

The antimicrobial potential of the PVA/CS electrospun nanofiber Films containing CFX-MUP was evaluated by the agar disc diffusion method [30] against pathogenic microbes used for this study. All formulations were evaluated for anti-bacterial activities against *Staphylococcus aureus*. The agar media was prepared by dissolving 0.0895g of the nutrient agar in 60 mL of deionized water. Both nutrient-broth and Petri dishes were autoclaved for 3h, and after that, 20mL of media was spread in each petri plate and solidified. Afterward, bacterial strains were spread uniformly on the agar media. Once the paper disc of each sample solution and nanofibrous discs were placed at predetermined sites, and plates were incubated at 37ºC for 24 hours. All the nanofibrous Films with varying cefadroxil and mupirocin concentrations were evaluated, while pure cefadroxil was utilized as a control. Yellow colonies were formed for *S. aureus.* after removing plates of the broth from an incubator. The zones of inhibition (ZoI) were measured by analyzing the mean diameter of inhibition of cefadroxil-mupirocin-loaded PVA/Chitosan nanofibers.

### 2.6 Statistical Test

The difference between different findings was assessed by applying One-way ANOVA, setting a level of significance at 0.05.

## 3 RESULTS AND DISCUSSION

### 3.1 Chemical Characterization of Nanofiber Films

#### 3.1.1 Scanning Electron Microscopy

**Fig. (2):**
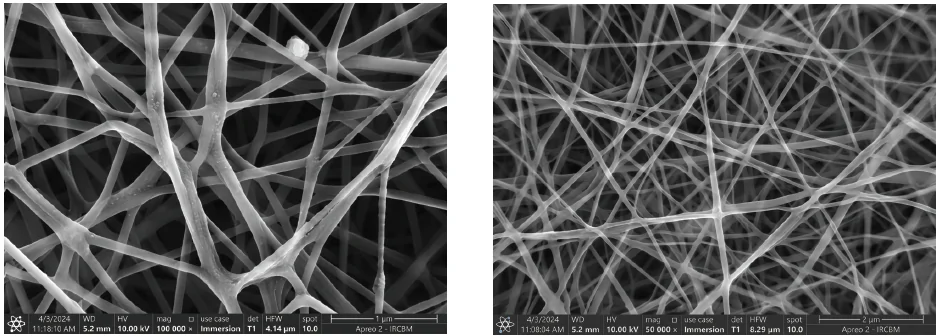
SEM images of PVA/CS and CFX-MUP loaded nanofibers.

SEM was employed to observe the surface morphological properties of MMT-reinforced PVA/CS drug-loaded and drug-free nanofiber films. The SEM photographs of both the CFX-MUP loaded and blank nanofibers were taken to compare and understand the overall diameter of the fibers (Fig. **2**). It was observed that there were no significant changes due to the drug loading of cefadroxil and mupirocin. The nanofiber morphology was uniform without the evidence of bead formation. There are enormous void spaces in the scanned films, indicating its ability to allow gaseous exchange and discharge/absorption of exudates, which is a prerequisite for wound healing [31].

#### 3.1.2 FTIR Spectra of Nanofibers

The FTIR spectroscopy of mupirocin, polymers, MMM-1, and MMM-4 electrospun nanofiber Films are presented in Fig. (**3**). This was carried out to determine the nature of the interactions that occurred between the functional groups of the various drugs and polymers. Due to the inclusion of MMT in PVA/CS nanofibers, the FTIR spectra showed multiple peaks at different wavenumber ranges and also depicted similar peaks for CFX-MUP incorporated electrospun nanofiber as of the pure mupirocin drug. The characteristic peaks that appeared at 3310 cm^-1^, 2935 cm^-1^, 1720 cm^-1,^ and 1084 cm^-1^ were assigned to O-H, C-H, C=O carbonyl and C-O-H stretching vibrations respectively [32]. Specifically, the shift in the wavenumbers of the O-H and C-H stretching bands occurred after both the drugs cefadroxil and mupirocin were incorporated in the PVA/CS nanofibers when compared to PVA, chitosan, cefadroxil, and mupirocin samples [33]. This demonstrates the ability of polymers like PVA and chitosan to effectively incorporate cefadroxil and mupirocin *via* electrospinning, indicating excellent biocompatibility between the polymers and drugs [34]. There was no appearance of new peaks when comparing the FTIR spectra of bare nanofibers with CFX-MUP-loaded nanofibers; this means that the functional groups did not interact at all.

#### 3.1.3 Thermal Gravimetric Analysis

The TGA thermogram of PVA/CS electrospun nanofibers, along with 1st-order derivative weight loss (%) with respect to temperature, is depicted in Fig. (**4**). For these formulations, three different degradation stages were observed. The TGA results of these samples depict various weight loss regions at different temperature ranges. The first weight loss occurred at around 40°C, which corresponds to the loss of water and solvent content present in the nanofibrous scaffold. The second weight loss was at about 100-130ºC, where the thermal decomposition of the nanofibrous scaffold occurred. In the third step, weight loss at 300ºC was observed because of the degradation of side chains of chitosan and PVA as depicted in the previous works [35-38]. Chitosan and PVA degradation at a temperature range of 370ºC were almost similar, and further weight loss at and above 370ºC was because of the drug content [36]. The TGA studies also reveal that the thermal stability of PVA/CS nanofibers has not been compromised due to the use of fillers. However, the TGA thermogram for CFX-MUP-loaded electrospun nanofibers indicated higher stability than drug-free nanofibers owing to the heat resistance characteristic exhibited by cefadroxil and mupirocin.

#### 3.1.4 XRD Spectroscopy

Structural characteristics of PVA/CS nanofibers reinforced with MMMT before and after drug loading were documented by X-ray diffraction analysis. Fig. (**5**) represents the X-ray diffractogram of the fabricated nanofibers, chitosan, and PVA. In M4, cefadroxil shows intense peaks between 2 of 10 and 25. The sharp peaks reveal the crystalline nature of cefadroxil after encapsulation in the nanofibers [37]. The sharp peaks were observed for the fabricated nanofibers indicating the crystalline nature of cefadroxil and mupirocin. The peaks were slightly shifted due to obstruction of inter and intra-molecular hydrogen bonding between PVA, chitosan, and drugs [38, 39].

### 3.2 Pharmaceutical studies

#### 3.2.1 Antimicrobial Activity of Nanofibers

The antimicrobial activity for CFX-MUP loaded PVA/CS nanofibers was performed against gram-positive bacteria *staphylococcus aureus*. All the formulations have been tested for anti-bacterial activity. The antibacterial results are displayed in Table **2**. The blank sample showed activity due to the presence of chitosan which has anti-bacterial properties, antioxidant and anti-inflammatory properties. The zone of inhibition (ZoI) measured for the blank sample was very least, *i.e.* 5 mm, which was because chitosan does not diffuse easily in the agar diffusion procedure as reported in a previous study [40]. MMM-2 showed ZoI of 10 mm which is due to the antibiotic cefadroxil. The formulation MMM-3 and MMM-4 showed a significantly highest (*p* < 0.05) ZoI (*i.e*., 20 mm), which is due to the presence of mupirocin in the nanofibrous scaffold in comparison with the control, which showed a ZoI of 15 mm and 17 mm respectively. The value of the diameter of ZOI for PVA/CS MP increases with increasing the amount of mupirocin due to its anti-bacterial properties. The formulation MMM-5 showed 10 mm of ZoI, which is half of the ZoI of MMM-3 and MMM-4. This is due to the antimicrobial activity of mupirocin, which is more than cefadroxil [41]. The results showed excellent activity in inhibiting the Gram-positive bacteria *Staphylococcus aureus* and its effectiveness for wound healing.

#### 3.2.2 In-vitro Drug Release Kinetics

The drug dissolution test was performed to evaluate the drug-releasing capability of electrospun nanofibers. The *in-vitro* drug release kinetics for both drugs is shown in Fig. (**6**). The formulations MMM-3, MMM-5, and MMM-6 showed an initial burst release of mupirocin. The formulation MMM-3 has pure mupirocin, and its burst release is higher than other formulations. Overall, MMM-3 showed a significantly (*p* < 0.05) higher rate of drug release compared to other formulations. All the formulations have a cumulative drug release of more than 50%. The cumulative drug release concentration of mupirocin for formulation MMM-6; 63.3%, MMM-5; 62.23%, MMM-4; 60.43%; MMM-3; 65.45% respectively. However, more than 80% of mupirocin was diffused out of the nanofibrous scaffolds after 2 h. The different chemical linkages in the nanofibrous scaffolds did not interfere with the release of mupirocin from the nanofibrous scaffolds [42]. Therefore, the drug dissolution test revealed that the release of mupirocin would be easily available at the site of action when it is applied to the burn wound.

Release rates were higher in formulations containing greater concentrations of cefadroxil in comparison to formulations having higher quantities of the same compound. A strong intermolecular network between PVA and chitosan contributes to the delayed release of therapeutic drugs from the scaffolds. Comparable results were presented in a previous study [43]. Furthermore, a study reported the regression coefficient R^2^, of which the Korsmeyer-Peppas model was the best to be fitted on all the formulations [44, 45].

#### 3.2.3 Wound Healing Activity of Fabricated Nanofibers

The wound healing study involved the use of MMM4 to assess its therapeutic potential since it exhibited the highest antibacterial effect, possibly due to the presence of drugs. The *in-vivo* study was performed to determine the relative extension of wounds over time. Wound healing of 20 days is depicted in Figs. (**7** and **8**) below.

**Fig. (8):**
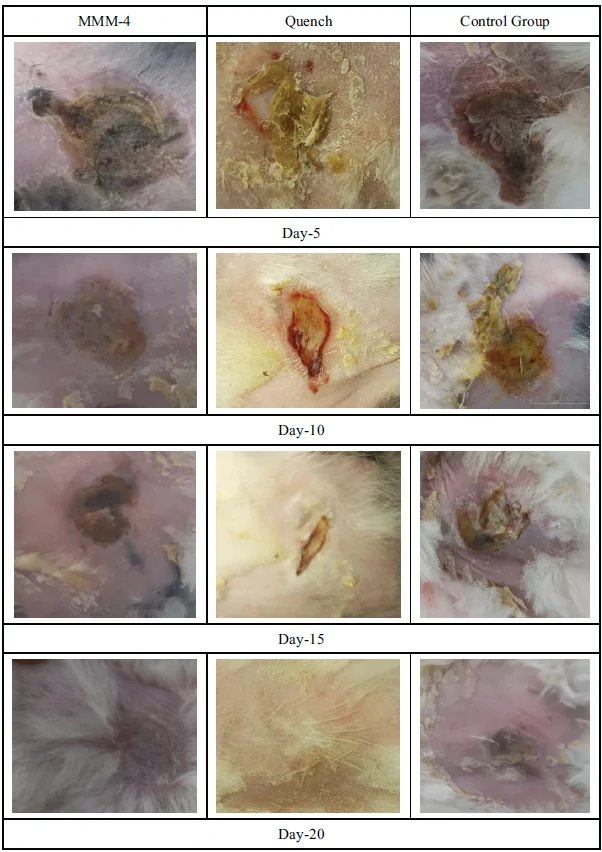
Macroscopic view for *in vivo* wound healing activity on different days.

The macroscopic evaluation results showed that the average wound healing of cefadroxil/mupirocin-loaded nanofibers was significantly (*p* < 0.05) higher than blank and market quench. The cefadroxil/mupirocin-treated nanofibers show 80% wound healing in 10 days. The wound contraction was higher in a formulation containing 0.15 g mupirocin. Measurement of treatment revealed that the formulation that contains the higher amount of mupirocin exhibited higher wound contraction as compared to other formulations for 20 days. On the basis of these findings, it can be expressed that this dual drug delivery system has a promising potential to efficiently treat infected wounds (Fig. **8**).

#### 3.2.4 Histopathological Studies

The efficacy of cefadroxil-mupirocin-loaded PVA/CS nanofibers for wound healing activity was assessed by histopathological studies. The photomicrographs at different stages of the wound healing process were taken by OLYMPUS-DP 72 digital camera. Fig. (**9**) presents the histopathological analysis of the control group, drug-loaded electrospun nanofibers, and drug-free nanofibers. The histopathological examination confirmed that the cefadroxil-mupirocin incorporated PVA/Chitosan nanofibers hold the maximum wound healing efficacy and re-epithelialization [46, 47]. The CFX-MUP-loaded PVA/CS nanofibers demonstrated more enhanced healing ability compared to drug-free nanofibers as well as the other groups. Nanofibers that have been incorporated with drugs showed better arrangement of collagenous fibers and thick layering of skin tissues as compared to other groups [48]. This can be attributed to the fact that nanofibers, when conjugated with drugs, possess better antibacterial and anti-inflammatory characteristics, which leads to faster healing of burn injuries [49].

## CONCLUSION

This study successfully developed and characterized dual drug-based PVA/chitosan nanofiber films reinforced with MMT for advanced burn wound management. The nanofiber exhibited a smooth appearance and considerable antibacterial activity, achieving inhibition zones of up to 20 mm. *In vitro* evaluation confirmed improved wound healing with about 90% wound repair by day 17 compared to untreated wounds. The hypothesis that dual drug loading of cefadroxil and mupirocin provides synergistic antibacterial impact and improved healing was validated. Compared to other nanofiber-wound dressings narrated in recent literature, our films showed better performance in the context of antibacterial efficacy. This innovation provides a potential alternative to conventional wound care, addressing the growing challenge of antibiotic resistance. Future studies will optimize polymeric blends for better mechanical features and conduct prolonged efficacy studies in larger animals to advance clinical uses.

## STUDY LIMITATIONS

The use of a small number of animals (three rabbits) in the *in vivo* wound healing assessment is a limitation of our study. The use of three rabbits in the *in vivo* wound healing study was based on ethical considerations aligned with the principles of the 3Rs (Replacement, Reduction, and Refinement) to minimize animal usage. This preliminary investigation aimed to assess the feasibility and efficacy of the drug-loaded nanofiber films, providing foundational data for future, larger-scale studies.

## Figures and Tables

**Fig. (3):**
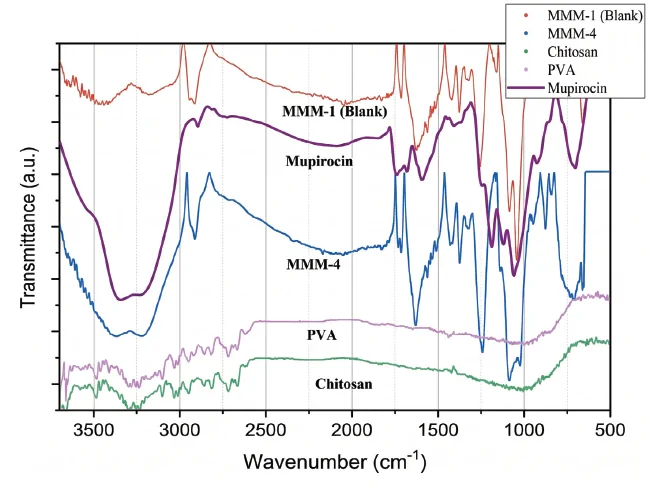
FTIR overlay of electrospun NFs, polymers and drug.

**Fig. (4):**
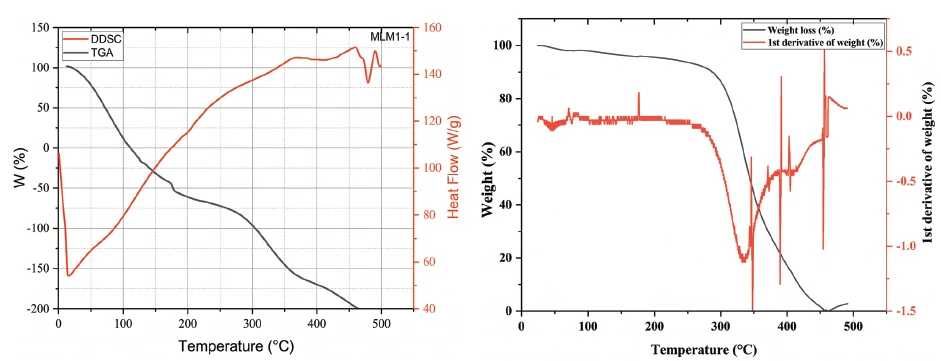
TGA thermogram for MMM-4 and 1^st^ order derivative of weight (%) loss.

**Fig. (5):**
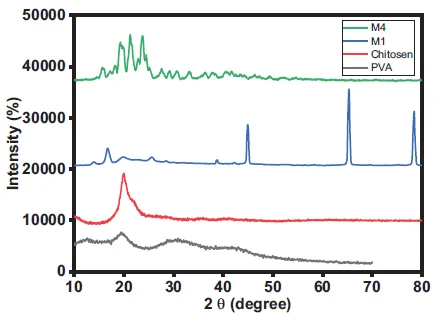
X-ray diffractograms of polymers, drug-loaded, and drug-free electrospun nanofibers.

**Fig. (7):**
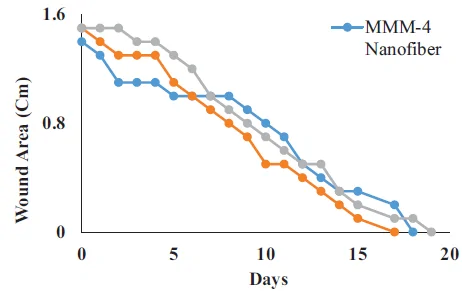
Wound healing study for various formulations.

**Fig. (6):**
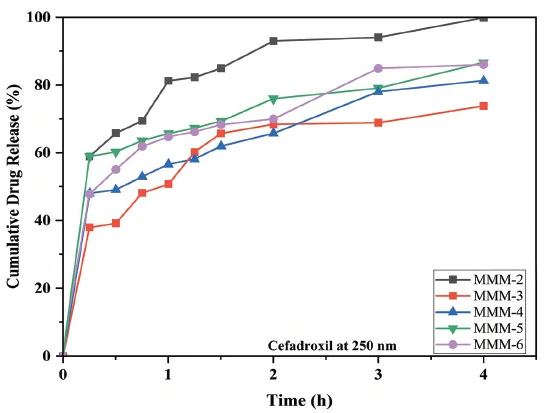
Drug release studies of drug-loaded nanofibers.

**Fig. (9):**
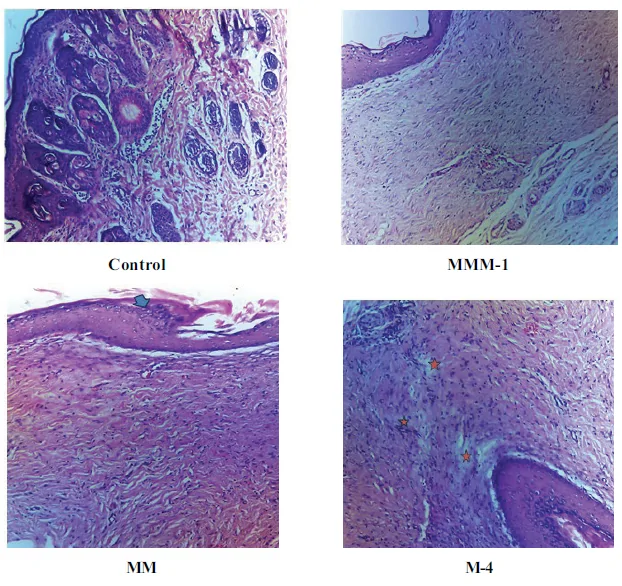
Histopathological results of control, MMM-1 and MMM-4 electrospun nanofibers.

**Table 1:** Composition of various formulations, each having a concentration of PVA solution = 10% by weight, chitosan solution = 2% by weight, and MMT = 1%.

**Formulation Code**	**Cefadroxil**	**Mupirocin**
MMM-1 (Blank)	0.0 g	0.0 g
MMM-2	0.2 g	0.0 g
MMM-3	0.0 g	0.2 g
MMM-4	0.1 g	0.1 g
MMM-5	0.15 g	0.50 g
MMM-6	0.50 g	2.15 g

**Table 2:** Zone of inhibition of all formulations of fabricated nanofibers.

**Sample (6mm)**	**Zone of Inhibition**		
**Formulation code**	***Staphylococcus aureus***	**Control Mupirocin**	**Control Cefadroxil**
MMM-1	5 mm		
MMM-2	10 mm		
MMM-3	20 mm	17 mm	15 mm
MMM-4	20 mm		
MMM-5	10 mm		
MMM-6	18 mm		

## Data Availability

The data and supportive information are available within the article.

## LIST OF ABBREVIATIONS

CS: Chitosan Solution
CUI: COMSATS University Islamabad
MMT: Montmorillonite
PVA: Polyvinyl Alcohol
ZoI: Zones of Inhibition
